# Supplementary material for: Rare truncating variants in the sarcomeric protein titin associate with familial and early-onset atrial fibrillation
Source: Nat Commun. 2018 Oct 17;9:4316. doi: 10.1038/s41467-018-06618-y (PMC6193003; doi:10.1038/s41467-018-06618-y)
Supplement: Supplementary file 1 — Supplementary Information [file 41467_2018_6618_MOESM1_ESM.pdf]

Supplementary Information to:

G. Ahlberg et al. **Rare truncating variants in the sarcomeric protein titin associate with familial and early-onset atrial fibrillation**

Table of Figures

Supplementary Figure 1. Inclusion of families 2
Supplementary Figure 2. MDS Exomes 3
Supplementary Figure 3. Batch effect analysis with MDS 4
Supplementary Figure 4. Relatedness Analysis 5
Supplementary Figure 5. MDS lone AF 6
Supplementary Figure 6. Coverage TTN exome sequencing. 7
Supplementary Figure 7. Distribution of identified rare TTNtv. 8
Supplementary Figure 8. Proteomics expression analysis of TTN 9
Supplementary Figure 9. Montage of Z-stack images, wild type (WT) Zebrafish 10
Supplementary Figure 10. Montage of Z-stack images, mutant zebrafish 11
Supplementary Figure 11. Defective z-discs in a zebrafish titin truncated homozygous mutant 12
Supplementary Figure 12. Sarcomere TEM images larval stage 13
Supplementary Figure 13. Sarcomere length and width in mutant vs. wildtype zebrafish 14

Table of Tables

Supplementary Table 1. Sequencing metrics exomes 15
Supplementary Table 2. Medication at inclusion 16
Supplementary Table 3. Sarcomere length ANOVA 17
Supplementary Table 4. Sarcomere width ANOVA 18
Supplementary Table 5. Zebrafish ECG parameters 19

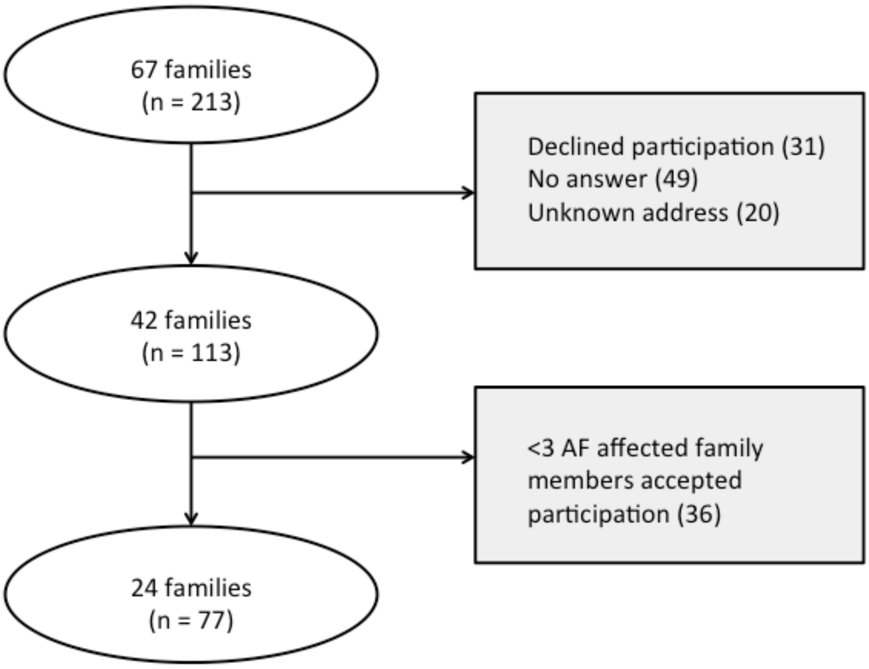

Supplementary Figure 1. Inclusion of families

Inclusion of families with ≥ 3 members with the diagnosis of atrial fibrillation. Number of individuals are shown in parenthesis.

a) b)

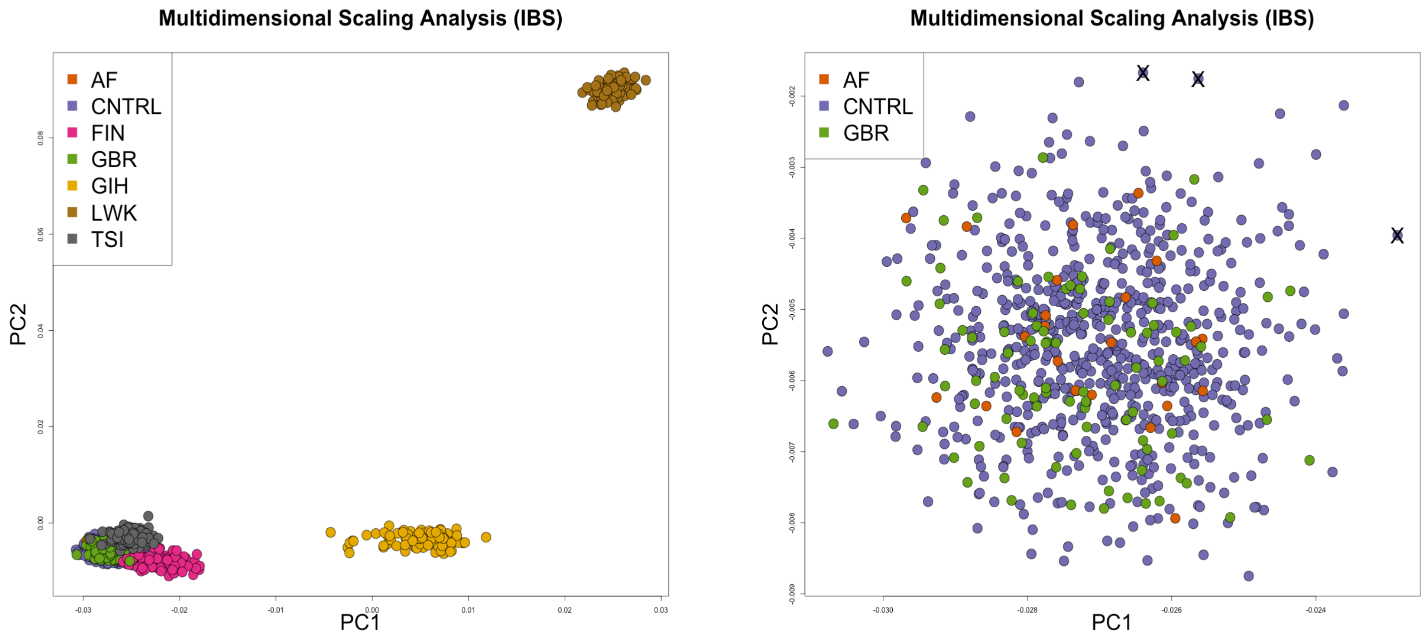

Supplementary Figure 2. MDS Exomes

MDS population structure inference of AF families and control group. Reference populations came from the 1000 genomes project's phase3 dataset. Population codes: AF families index patients (AF), control group (CNTRL), Finish in Finland (FIN), British in Britain and Scotland (GBR), Luhya in Kenya (LWK), Toscani in Italy (TSI), Gujarati Indian from Texas (GIH). a) Principal Components 1 and 2, showing study population and populations from Europe, India, and Africa. b) Plot showing the same Principal Components as in figure A, zoomed in on region with AF, CNTRL, FIN, and GBR population. 'X's indicates excluded samples

a)

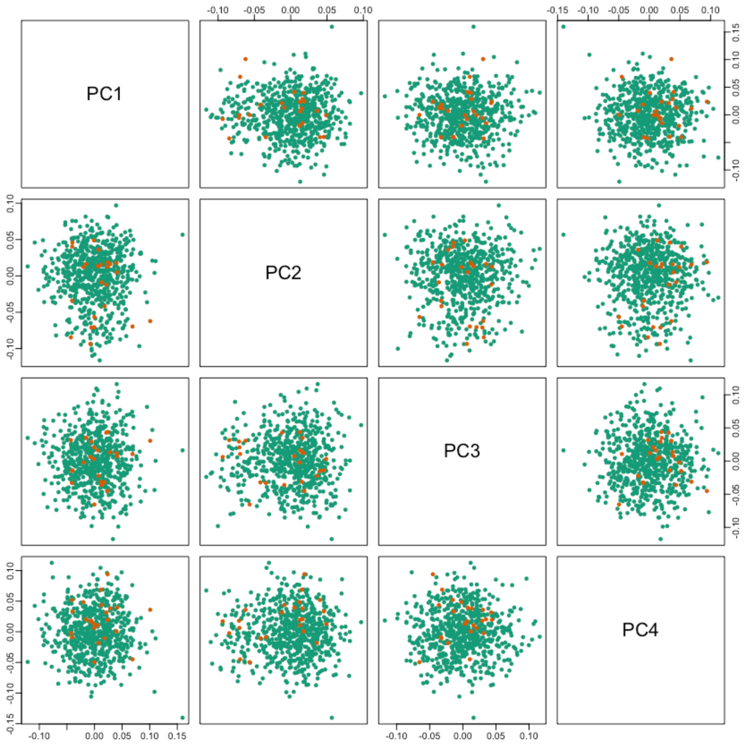

b)

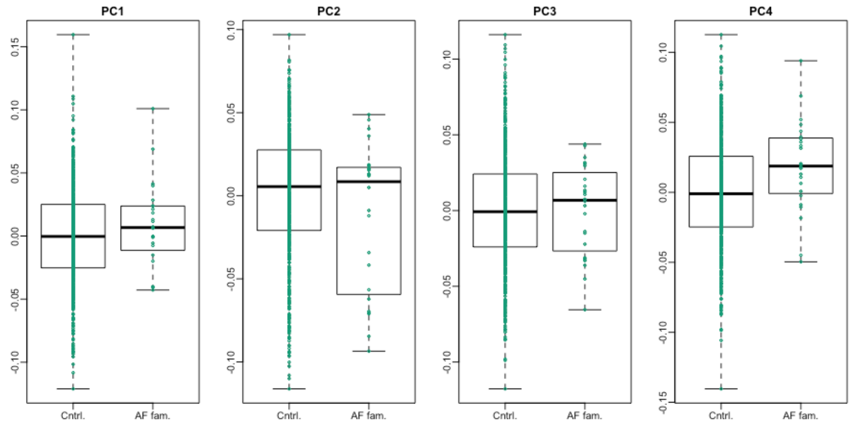

Supplementary Figure 3. Batch effect analysis with MDS

Exploratory data analysis of batch effect with MDS. a) Plots of AF index patients and control group samples in principal components (PC) 1-4. b) Boxplots of PC 1-4, with median, interquartile ranges (IQR), and whiskers with upper/lower QR +/- 1.5*IQR, in AF index patients (n=24) and control group (n=663).

a)

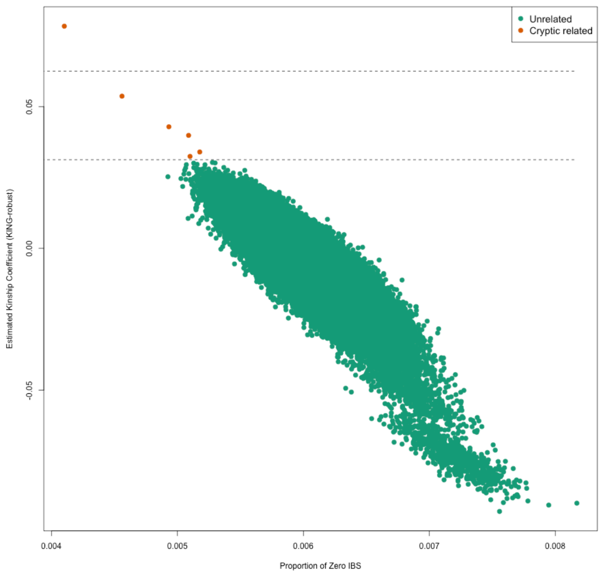

b)

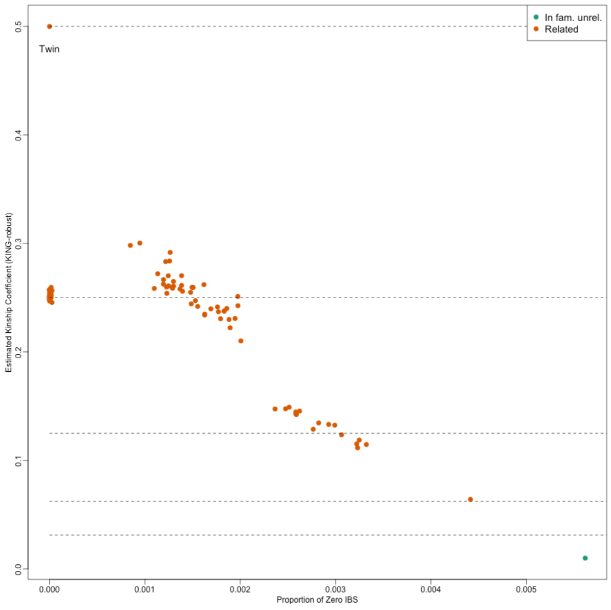

Supplementary Figure 4. Relatedness Analysis

Relationship inference using King-robust algorithm of filtered and LD pruned dataset. Kinship coefficient (KING) is shown on y-axis, proportion of Zero IBS on x-axis. Each dot represents relatedness of one pair. a) Showing pairs of all stated unrelated pairs. Pairs with a kinship coefficient >1/32 is tagged as cryptic related b) Showing all pairs within respective AF family. In family but unrelated are tagged as “In fam. unrelated”. There was one pair in one AF family that were unrelated; this was in agreement with the kinship coefficient

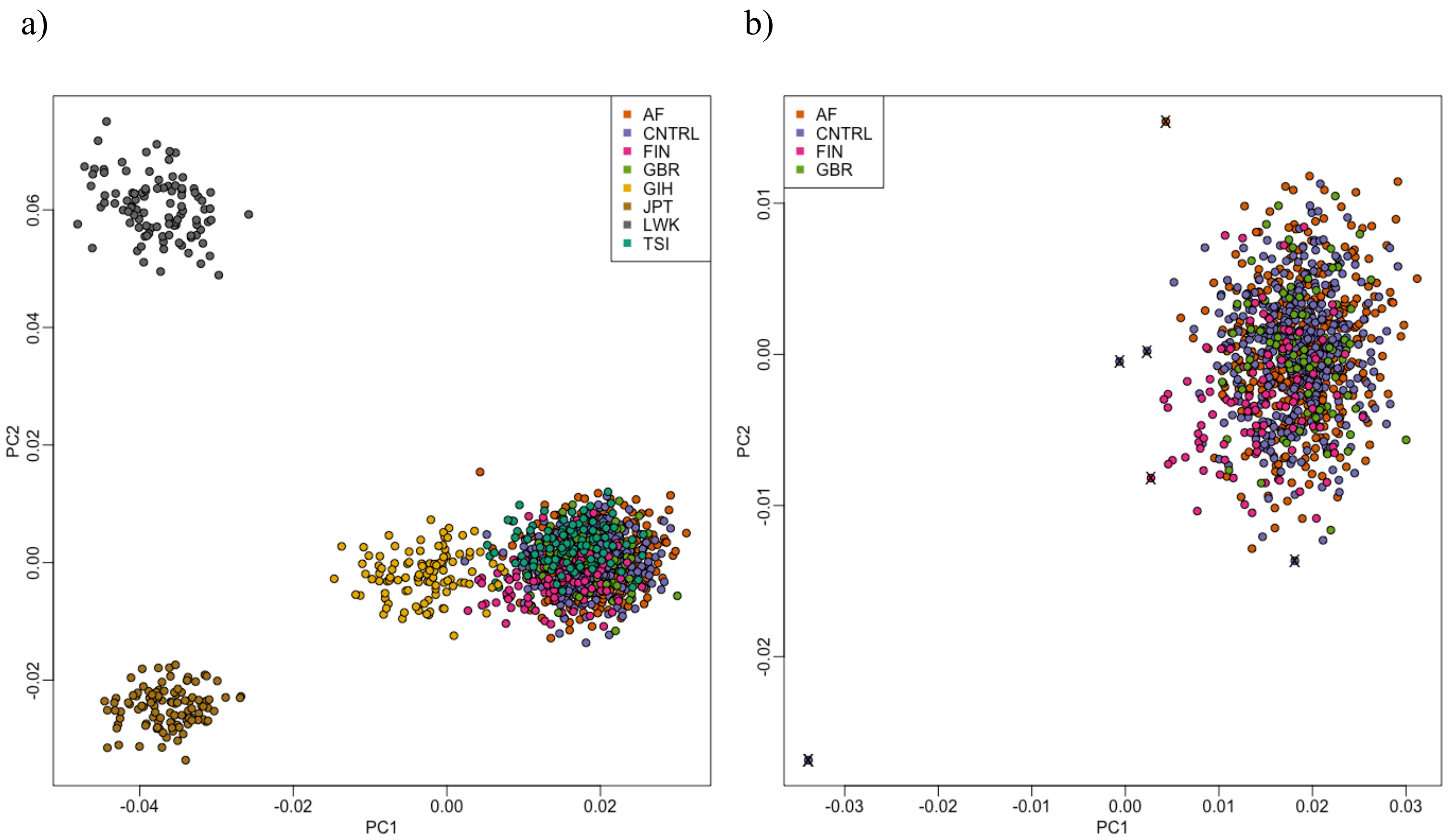

Supplementary Figure 5. MDS lone AF

MDS population structure inference of lone AF cohort. Reference populations came from the 1000 genomes project’s phase3 dataset. Population codes: Lone AF subjects (AF), Control group B (CNTRL), Finish in Finland (FIN), British in Britain and Scotland (GBR), Luhya in Kenya (LWK), Toscani in Italy (TSI), and Gujarati Indian from Texas (GIH). a) Principal Components 1 and 2, showing study population and populations from Europe, India, and Africa. b) Plot showing the same Principal Components as in figure A, zoomed in on region with AF, FIN, and GBR populations. ’X’’s indicates ethnic outliers and excluded samples.

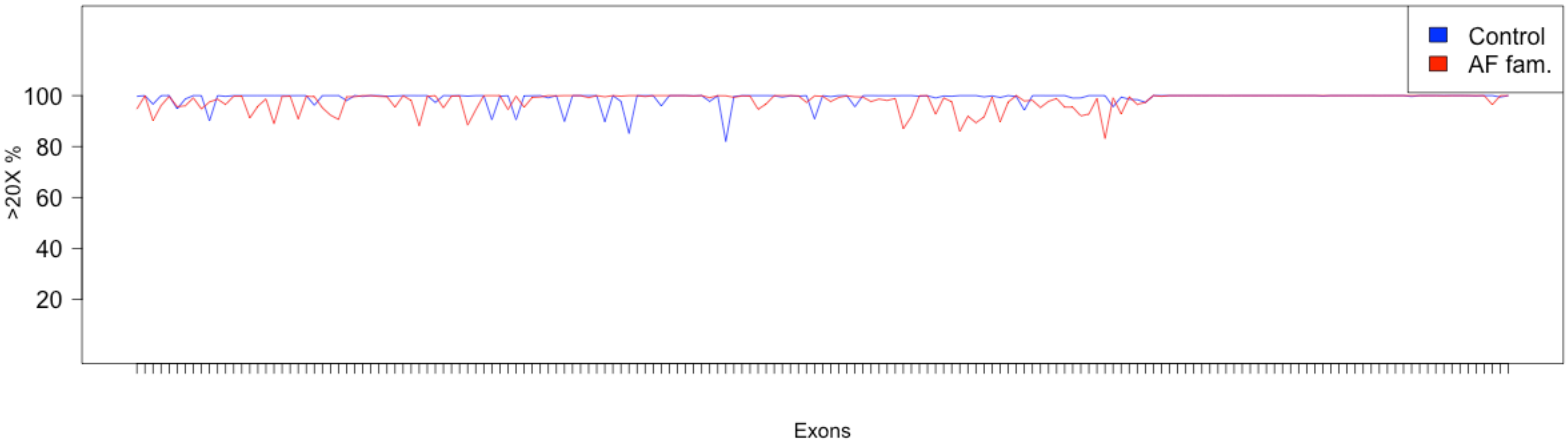

Supplementary Figure 6. Coverage TTN exome sequencing.

Coverage plot in exomes for *TTN* exons (PSI[1] > 90). The mean value of percentage of exon region over >20X is shown on Y-axis and exons in the dataset are shown on X-axis. The blue line represents the control group A (n = 663) and the red line represents the AF families group (n = 77).

[1] PSI - percent spliced in: a measure of splicing derived from RNAseq, this is an estimate of the percentage of TTN transcripts that incorporate into a given exon.

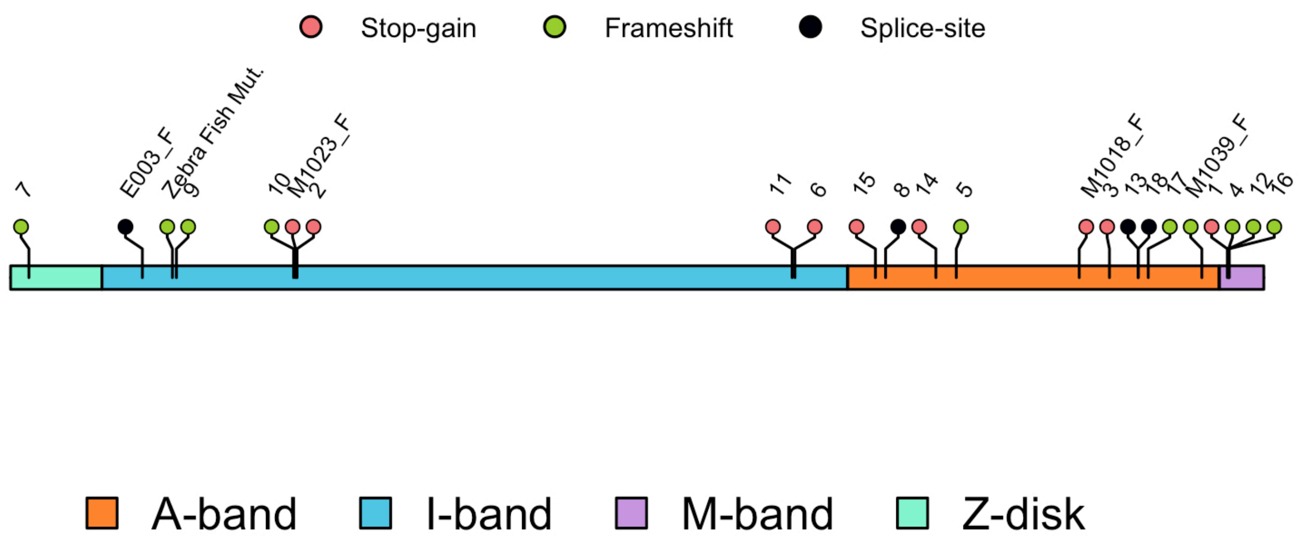

Supplementary Figure 7. Distribution of identified rare TTNtv.

TTNtv in principal cardiac long isoform ENST00000591111 identified in families are denoted with "_F"

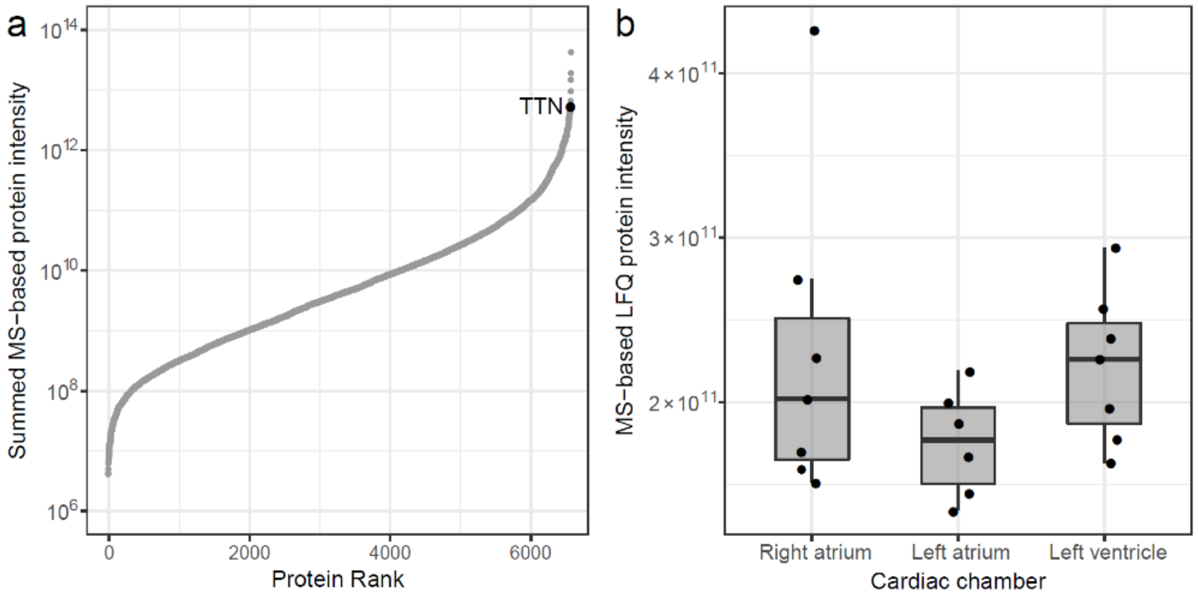

Supplementary Figure 8. Proteomics expression analysis of TTN

Expression level of titin evaluated by mass spectrometry (MS)-based protein intensity measurements from human heart biopsies from seven patients. a) 6,588 cardiac proteins were ranked based on MS intensity. Global intensity-ranked protein expression profile found TTN to be the 10th most abundant protein in human heart. b) Comparison of the relative protein expression level of TTN in atrial and ventricle biopsies using label-free quantification (LFQ) of the measured protein intensities. Individual measurements are depicted as black dots, boxplots show 25th, 50th, and 75th percentile of the distribution (interquartile range (IQR), box), and 1.5×IQR (whiskers). There is no statistically significant difference according to two sample t-test ($p>0.05$).

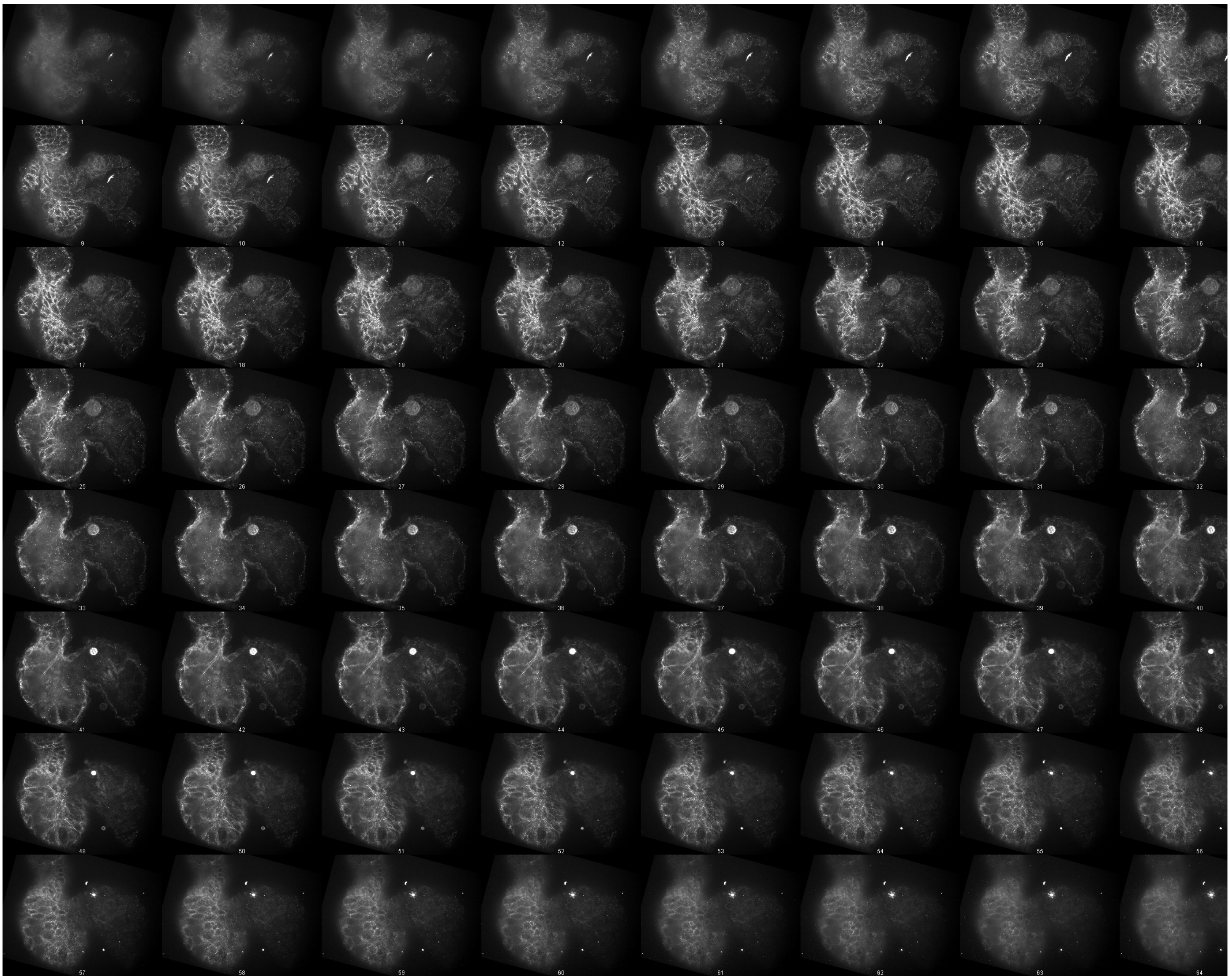

Supplementary Figure 9. Montage of Z-stack images, wild type (WT) Zebrafish

Complete Z-stack images of the WT zebrafish hearts shown in the manuscript's figure 3

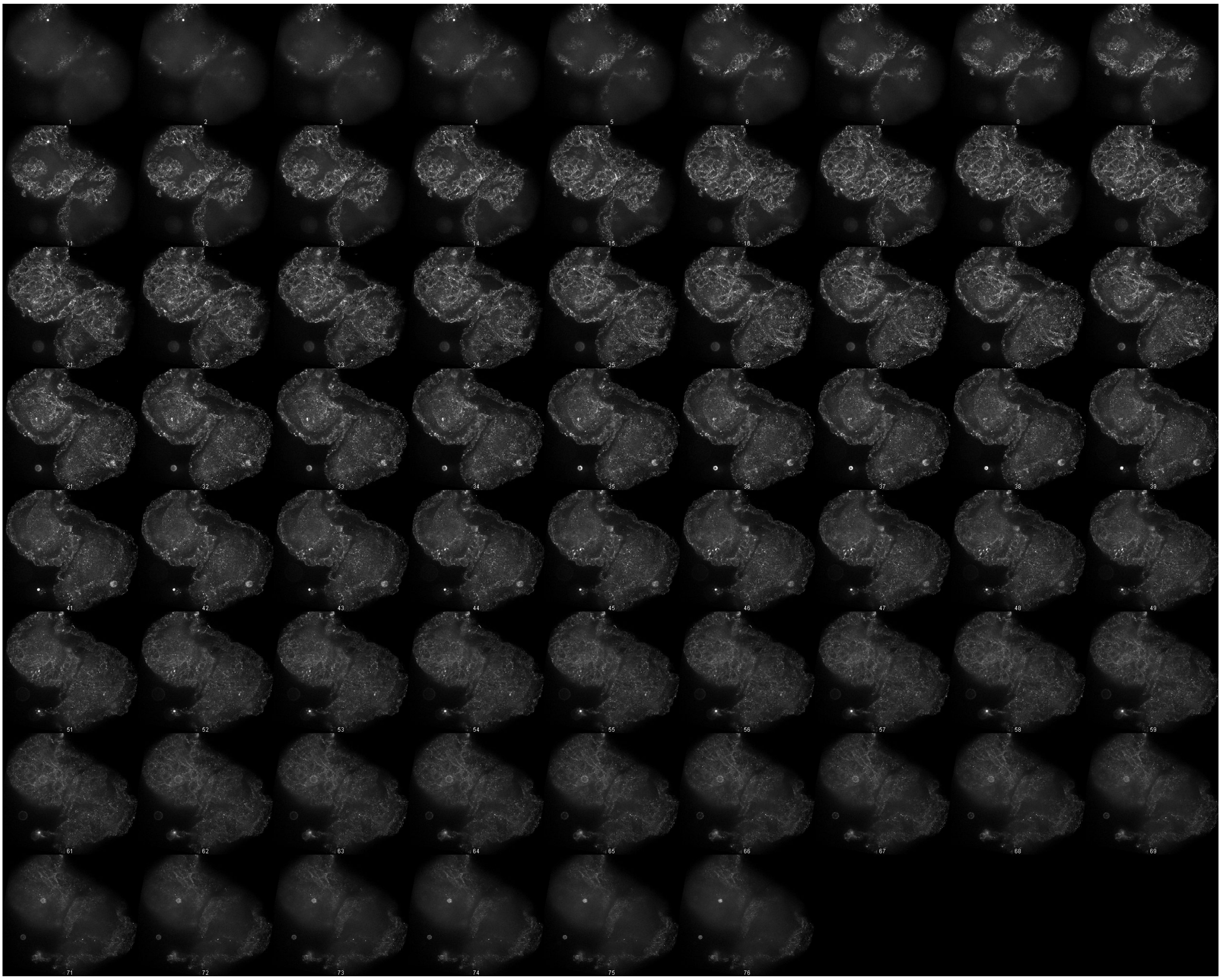

Supplementary Figure 10. Montage of Z-stack images, mutant zebrafish

Complete Z-stack images of the mutant zebrafish hearts shown in the manuscript's figure 3

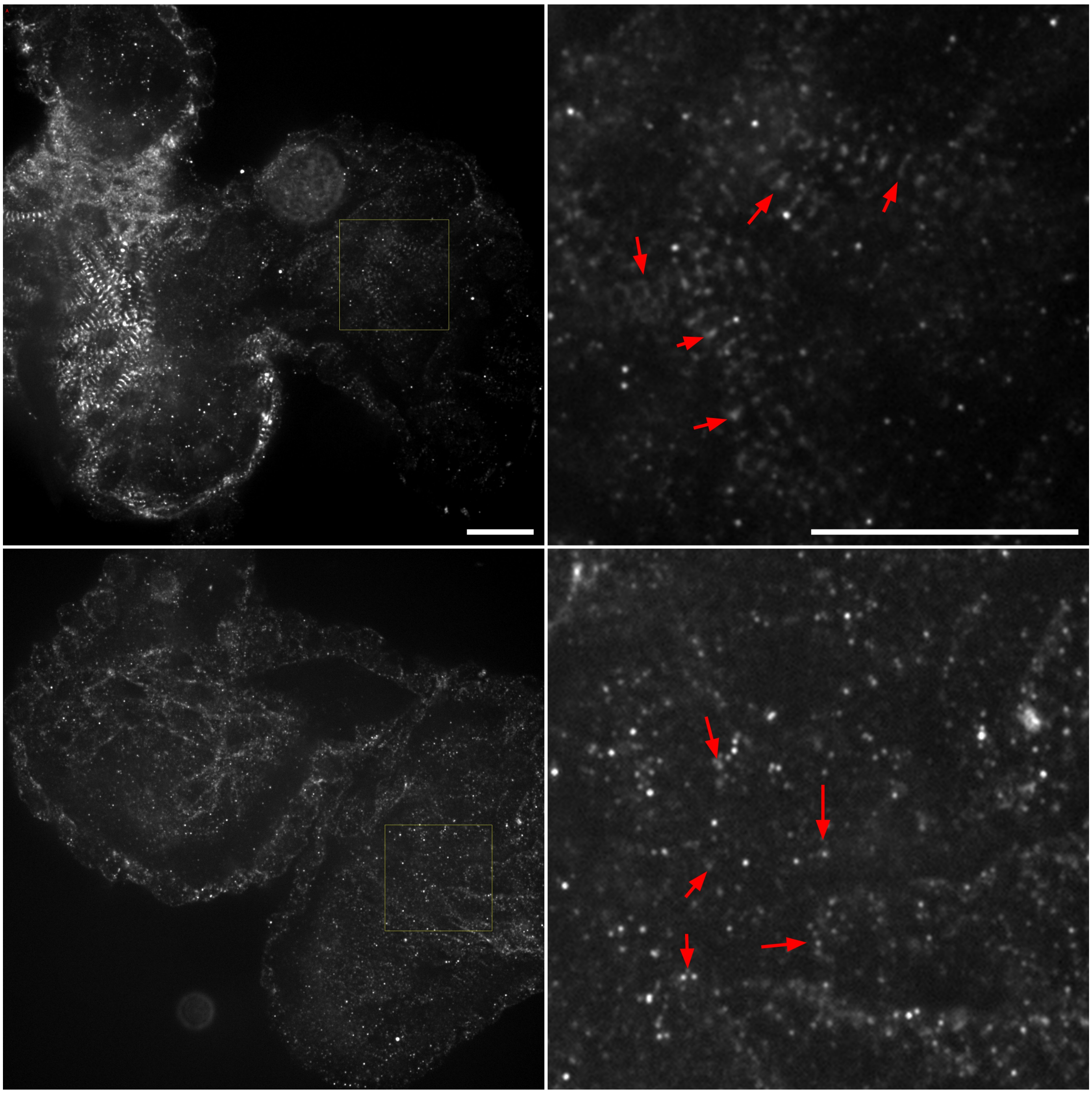

Supplementary Figure 11. Defective z-discs in a zebrafish titin truncated homozygous mutant

Top panel) Isolated hearts form 72 hours post fertilization old zebrafish stained for alpha-actinin, an important component of the Z-disc, revealed well-defined z-discs in both the atria (right chamber) and ventricle (left chamber) in the WT siblings. A close up of the atria (right) show well-defined z-discs (red arrows). **Bottom panel**) Compared to the observations made in the isolated hearts from the WT sibling embryos, the homozogous mutant embryos showed a no well-defined z-discs, and an aberrant alpha-actinin expression in the atria (indicated by the red arrows in the bottom right image) and the ventricle indicating a compromised sarcomere architecture. Scale bars = 20µm. Please see supplementary Figure 9-10 for complete z-stacks.

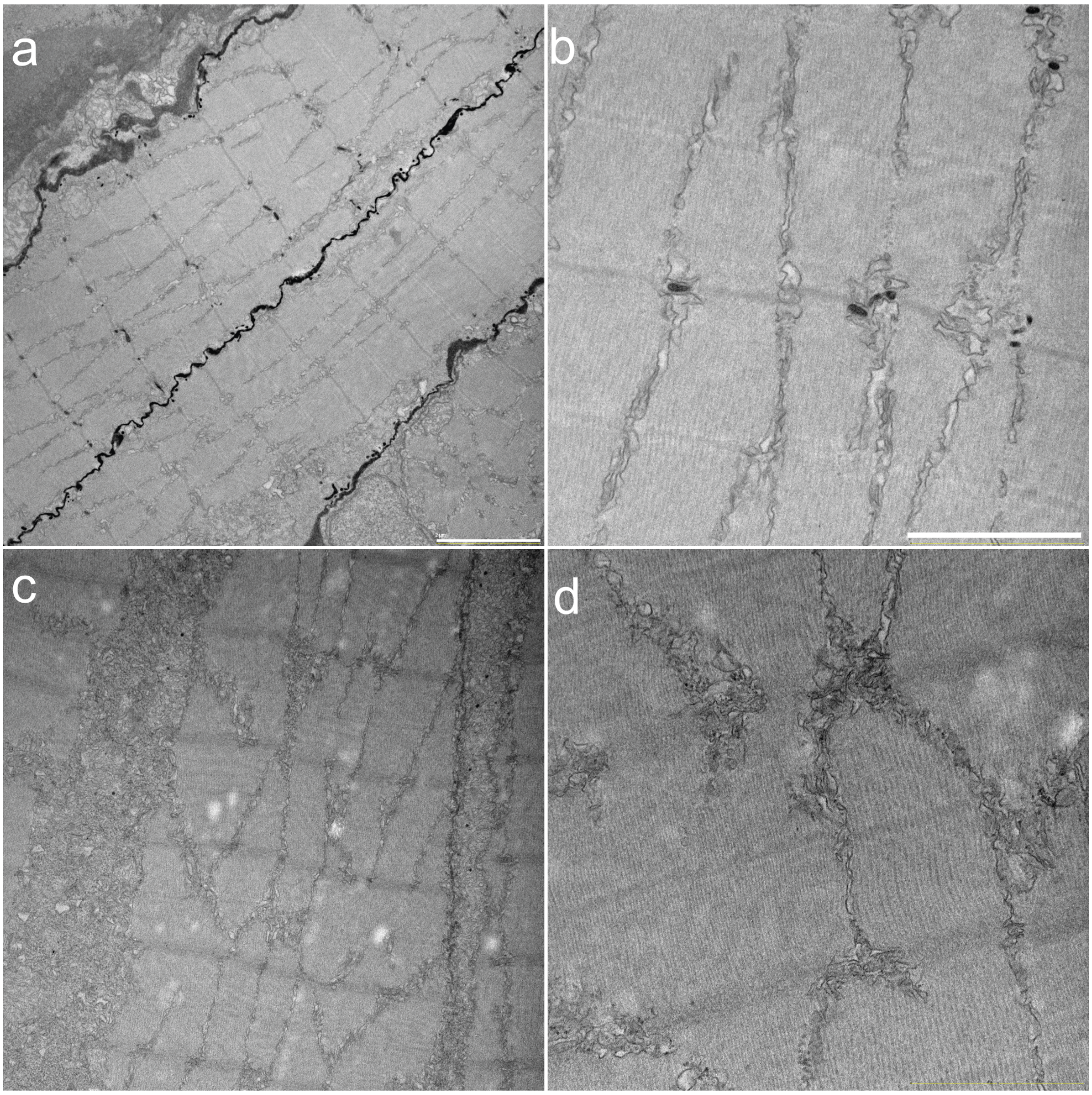

Supplementary Figure 12. Sarcomere TEM images larval stage

TEM images from 3 dpf *ttn.2*$^{sfc9+/+}$ (**a-b**) *and ttn2.*$^{sfc9+/-}$ (**c-d**) larvae hearts. **a)** show well-defined sarcomeres, with distinguishable Z-discs with adjacent I-bands, and distinct M-lines throughout the cardiac tissue. Scale bar 2µM (**a**)and 4 µM (**b**). In heterozygous mutant larvae (**c**) the sarcomere structure is less organised. The z-discs appear blurred and the I-bands are absent (**d**) Scale bar 2µM (**c**) and 4 µM respectively (**d**).

a)

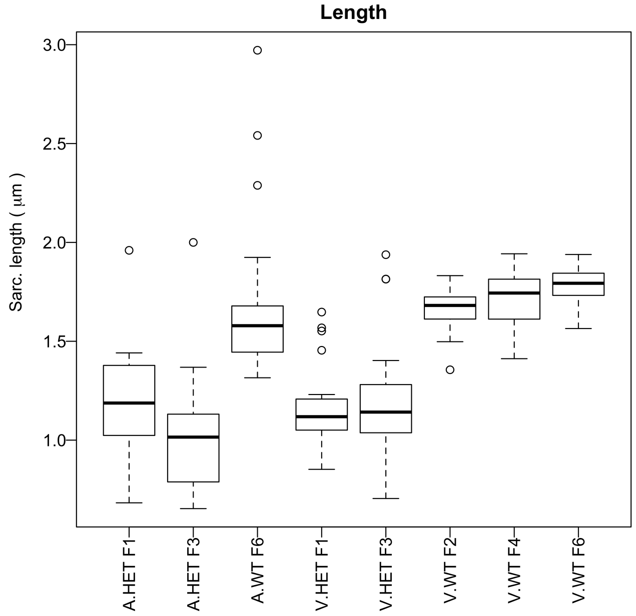

b)

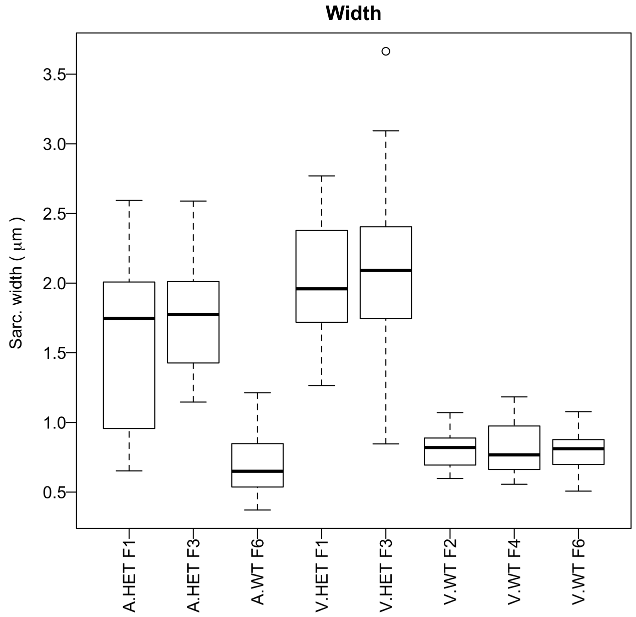

Supplementary Figure 13. Sarcomere length and width in mutant vs. wildtype zebrafish

Distribution of measured sarcomere length and width from TEM images and ImageJ, using the Feret function in ImageJ. Boxes are divided by zebrafish (F1-6), heterozygote (HET), wildtype (WT), atria (A.) and ventricular (V.). **a**) Measured length of sarcomere. **b**) Measured width of sarcomere.

Supplementary Table 1. Sequencing metrics exomes

Sequencing sample metrics with TiTv ratio/sample, number of alternative calls (N. Alt), and call rate. Metrics is shown by groups of samples in AF families and control samples.

	TiTv ratio/sample		N. Alt		Call rate (%)	
	AF families	Control group A	AF families	Control group B	AF families	Control group B
**Mean**	3.35	3.38	15,177	15,172	99.1	99.5
**Median**	3.35	3.37	15,182	15,175	99.1	99.6
**SD**	0.066	0.060	117	155	0.002	0.003

AF = atrial fibrillation; SD = standard deviation; TiTv = Transition to Transversion ratio

Supplementary Table 2. Medication at inclusion

Family	Pedigree ID	Medication at inclusion*
E003	II-2	Warfarin 2.5 mg (Dosage adjustment based on INR), Bisoprolol 2.5 mg x 1, Flecainid 100 mg x 1
E003	II-5	Unknown hypertensiva 10+5mg x 1, Warfarin 2.5 mg (Dosage adjustment based on INR), Thiamazol 2.5 mg x1, Digoxin 62.5mikrog x 2, Furosemid 40 mg x1
E003	III-4	Perindopril 5 mg x1. Metoprolol 200 mg x1. Eplerenon 25mg x 2. Potassium chloride 750 mg x 1. Furosemid 40 mg x 2
M1023	II-4	Digoxin 62.5 mg x 5, Carvedilol 12.5 mg x 2, Ramipril 5 mg x 2, Aspirin 75 mg x 1, Warfarin 2.5 mg (Dosage adjustment based on INR)
M1023	III-1	No medication at inclusion
M1023	III-3	Apixaban 5 mg x 2, Spironolacton 25 mg x 1, Ramipril 5 mg x2, Furosemid 40 mg x 3, Potassium chloride 750 mg x 3, Metoprolol 50 mg x2
M1018	III-1	Metoprololsuccinat 150 mg x 1, Simvastatin 40 mg x 1, Hydrochlorthiazid/Losartan 50/12.5 mg x 1, Digoxin 250 mikrog x 1, Warfarin 2.5 mg (Dosage adjustment based on INR)
M1018	IV-2	Hydrochlorthiazid/Losartan 50 mg/12,5 mg x 1
M1018	IV-3	No medication at inclusion
M1039	III-2	No medication at inclusion
M1039	III-3	No medication at inclusion
M1039	III-5	No medication at inclusion
M1030	III-4	Aspirin 150 mg x 1
M1030	III-7	Warfarin 2.5 mg (Dosage adjustment based on INR)
M1030	IV-5	No medication at inclusion

*Self-reported information at the time of inclusion.

Supplementary Table 3. Sarcomere length ANOVA

Analysis of variance in measured sarcomere length. Comparing fish 1-6 (F1-6), measurements from atrium (A) and ventricle (V), heterozygote (HET) and wildtype (WT).

Fish vs. Fish	Diff.	95% CI	P adj.
A.HET F3 - A.HET F1	-0,1662	-0.368 - 0.0355	0,1921
A.WT F6 - A.HET F1	0,4428	0.2629 - 0.6226	<0,0001
V.HET F1 - A.HET F1	-0,0204	-0.2264 - 0.1855	1
V.HET F3 - A.HET F1	-0,0155	-0.192 - 0.1609	1
V.WT F2 - A.HET F1	0,4697	0.275 - 0.6645	<0,0001
V.WT F4 - A.HET F1	0,5164	0.3313 - 0.7014	<0,0001
V.WT F6 - A.HET F1	0,583	0.3721 - 0.7939	<0,0001
A.WT F6 - A.HET F3	0,609	0.4315 - 0.7865	<0,0001
V.HET F1 - A.HET F3	0,1458	-0.0581 - 0.3498	0,3639
V.HET F3 - A.HET F3	0,1507	-0.0234 - 0.3248	0,1447
V.WT F2 - A.HET F3	0,636	0.4433 - 0.8286	<0,0001
V.WT F4 - A.HET F3	0,6826	0.4998 - 0.8653	<0,0001
V.WT F6 - A.HET F3	0,7492	0.5403 - 0.9582	<0,0001
V.HET F1 - A.WT F6	-0,4632	-0.6456 - -0.2809	<0,0001
V.HET F3 - A.WT F6	-0,4583	-0.6065 - -0.3101	<0,0001
V.WT F2 - A.WT F6	0,027	-0.1427 - 0.1966	0,9997
V.WT F4 - A.WT F6	0,0736	-0.0847 - 0.2319	0,8467
V.WT F6 - A.WT F6	0,1402	-0.0477 - 0.3282	0,308
V.HET F3 - V.HET F1	0,0049	-0.1741 - 0.1839	1
V.WT F2 - V.HET F1	0,4902	0.2931 - 0.6873	<0,0001
V.WT F4 - V.HET F1	0,5368	0.3493 - 0.7242	<0,0001
V.WT F6 - V.HET F1	0,6034	0.3904 - 0.8165	<0,0001
V.WT F2 - V.HET F3	0,4853	0.3193 - 0.6513	<0,0001
V.WT F4 - V.HET F3	0,5319	0.3775 - 0.6863	<0,0001
V.WT F6 - V.HET F3	0,5986	0.4139 - 0.7832	<0,0001
V.WT F4 - V.WT F2	0,0466	-0.1285 - 0.2217	0,9922
V.WT F6 - V.WT F2	0,1133	-0.089 - 0.3155	0,6792
V.WT F6 - V.WT F4	0,0667	-0.1262 - 0.2595	0,9648

Supplementary Table 4. Sarcomere width ANOVA

Analysis of variance in measured sarcomere wodth. Comparing fish 1-6 (F1-6), measurements from atrium (A) and ventricle (V), heterozygote (HET) and wildtype (WT).

Fish vs. Fish	Diff.	95% CI	P adj.
A.HET F3 - A.HET F1	0,2034	-0.1222 - 0.5289	0,5453
A.WT F6 - A.HET F1	-0,8962	-1.1835 - -0.6088	<0,0001
V.HET F1 - A.HET F1	0,4657	0.1367 - 0.7948	0,0006
V.HET F3 - A.HET F1	0,5068	0.2249 - 0.7887	<0,0001
V.WT F2 - A.HET F1	-0,7738	-1.085 - -0.4626	<0,0001
V.WT F4 - A.HET F1	-0,7629	-1.0585 - -0.4673	<0,0001
V.WT F6 - A.HET F1	-0,7693	-1.1063 - -0.4323	<0,0001
A.WT F6 - A.HET F3	-1,0995	-1.3869 - -0.8122	<0,0001
V.HET F1 - A.HET F3	0,2624	-0.0667 - 0.5915	0,228
V.HET F3 - A.HET F3	0,3034	0.0215 - 0.5854	0,025
V.WT F2 - A.HET F3	-0,9772	-1.2884 - -0.666	<0,0001
V.WT F4 - A.HET F3	-0,9663	-1.2618 - -0.6707	<0,0001
V.WT F6 - A.HET F3	-0,9727	-1.3096 - -0.6357	<0,0001
V.HET F1 - A.WT F6	1,3619	1.0706 - 1.6533	<0,0001
V.HET F3 - A.WT F6	1,403	1.1662 - 1.6398	<0,0001
V.WT F2 - A.WT F6	0,1224	-0.1486 - 0.3933	0,8653
V.WT F4 - A.WT F6	0,1333	-0.1196 - 0.3862	0,7429
V.WT F6 - A.WT F6	0,1269	-0.1733 - 0.4271	0,9011
V.HET F3 - V.HET F1	0,041	-0.245 - 0.327	0,9999
V.WT F2 - V.HET F1	-1,2396	-1.5544 - -0.9247	<0,0001
V.WT F4 - V.HET F1	-1,2286	-1.5281 - -0.9292	<0,0001
V.WT F6 - V.HET F1	-1,235	-1.5754 - -0.8946	<0,0001
V.WT F2 - V.HET F3	-1,2806	-1.5458 - -1.0154	<0,0001
V.WT F4 - V.HET F3	-1,2697	-1.5164 - -1.023	<0,0001
V.WT F6 - V.HET F3	-1,2761	-1.5711 - -0.981	<0,0001
V.WT F4 - V.WT F2	0,0109	-0.2688 - 0.2906	1
V.WT F6 - V.WT F2	0,0045	-0.3186 - 0.3277	1
V.WT F6 - V.WT F4	-0,0064	-0.3145 - 0.3017	1

Supplementary Table 5. Zebrafish ECG parameters

ECG Parameter	WT siblings ($ttn.2^{sfc9+/+}$) (ms)	Heterozygous siblings ($ttn.^{sfc9+/-}$) (ms)	P
**RR interval**	505.3 ± 29.6	560.1 ± 75.83	0.4927
**P-wave duration**	21.95 ± 0.7932	20.31 ± 2.782	0.5593
**PR interval**	48.49 ± 2.251	64.26 ± 2.653	**0.0005**
**QRS interval**	48.92 ± 1.434	51.78 ± 5.748	0.6186

ms = milliseconds
