# Supplementary material for: Rare truncating variants in the sarcomeric protein titin associate with familial and early-onset atrial fibrillation
Source: Nat Commun. 2018 Oct 17;9:4316. doi: 10.1038/s41467-018-06618-y (PMC6193003; doi:10.1038/s41467-018-06618-y)
Supplement: Supplementary file 3 — Description of Additional Supplementary Files [file 41467_2018_6618_MOESM3_ESM.pdf]

## Description of Additional Supplementary Files

File Name: Supplementary Data 1
Description: Rare loss of function variants in families.

File Name: Supplementary Data 2
Description: Clinical information on TTNtv carriers in the early-onset lone AF cohort.
